# Linkage analysis of alcohol dependence using MOD scores

**DOI:** 10.1186/1471-2156-6-S1-S162

**Published:** 2005-12-30

**Authors:** Konstantin Strauch, Robert Fürst, Franz Rüschendorf, Christine Windemuth, Johannes Dietter, Antonia Flaquer, Max P Baur, Thomas F Wienker

**Affiliations:** 1 Institute for Medical Biometry, Informatics, and Epidemiology, University of Bonn, Sigmund-Freud-Strasse 25, 53105 Bonn, Germany; 2 Bioinformatics and Gene Mapping Center, Max-Delbrück Center for Molecular Medicine, Robert-Rössle-Strasse 10, 13092 Berlin, Germany; 3 Institute of Medical Biometry and Epidemiology, Philipps University Marburg, Bunsenstrasse 3, 35033 Marburg, Germany

## Abstract

Alcohol dependence is a typical example of a complex trait that is governed by several genes and for which the mode of inheritance is unknown. We analyzed the microsatellite markers and the Affymetrix single-nucleotide polymorphisms (SNPs) for a subset of the Collaborative Study on the Genetics of Alcoholism family sample, 93 pedigrees of Caucasian ancestry comprising 919 persons, 390 of whom are affected according to DSM III-R and Feighner criteria. In particular, we performed parametric single-marker linkage analysis using MLINK of the LINKAGE package (for the microsatellite data), as well as multipoint MOD-score analysis with GENEHUNTER-MODSCORE (for the microsatellite and SNP data). By use of two liability classes, different penetrances were assigned to males and females. In order to investigate parent-of-origin effects, we calculated MOD scores under trait models with and without imprinting. In addition, for the microsatellite data, the MOD-score analysis was performed with sex-averaged as well as sex-specific maps. The highest linkage peaks were obtained on chromosomes 1, 2, 7, 10, 12, 13, 15, and 21. There was evidence for paternal imprinting at the loci on chromosomes 2, 10, 12, 13, 15, and 21. A tendency to maternal imprinting was observed at two loci on chromosome 7. Our findings underscore the fact that an adequate modeling of the genotype-phenotype relation is crucial for the genetic mapping of a complex trait.

## Background

Alcohol dependence occurs in many populations; it represents a complex trait with clear familial aggregation. It is more common in males than in females, and, in addition to social and psychological gender differences, genetic factors are supposed to act in a sex-specific way. In addition, genomic imprinting, which is also called parent-of-origin-effect, is assumed to play a role.

Here, we focus on the microsatellite and the Affymetrix single-nucleotide polymorphism (SNP) markers typed for the Collaborative Study on the Genetics of Alcoholism (COGA) family sample [1]. A set of 143 affected multigenerational pedigrees with 1,614 persons was provided for the Genetic Analysis Workshop 14 (GAW14). As with most complex traits which are governed by several genes, the disease model is unknown for alcohol dependence. Therefore, we took the approach of parametric exploratory linkage analysis, and performed single-marker LOD-score as well as multipoint MOD-score analysis for the dichotomous trait alcohol dependence. In a MOD-score analysis, the LOD score is maximized not only over the genetic position of the putative trait locus, but also with respect to the parameters of the disease model, i.e., the penetrances and the disease allele frequency [2].

Because the prevalence of alcoholism is higher in males than in females, it can be expected that, on average, the penetrances of a particular susceptibility locus are higher in males as well. Hence, we used separate liability classes for males and females. In order to investigate the role of parent-of-origin effects, we calculated MOD scores under models with and without imprinting. In addition, for the microsatellite data, we performed linkage analyses by using sex-averaged as well as sex-specific genetic maps.

## Data and Methods

### Dataset and phenotype

We focused on the 93 Caucasian pedigrees of the COGA dataset (including families with at most three founders without information on ethnicity). This subset consists of 468 males (50.9%) and 451 females (49.1%). We looked at the phenotype ALDX1, which is based on DSM-III-R [3] and Feighner criteria [4]. With this trait definition, there were 390 or 42.4% affecteds (69.2% males, 30.8% females) and 149 or 16.2% unaffecteds (18.1% males, 81.9% females). We assumed a person who never drank or showed inconclusive symptoms of alcohol dependence to be of unknown affection status. The pedigrees of the dataset were ascertained through 70 male and 21 female probands.

### Microsatellite data

We performed parametric exploratory linkage analysis with 315 microsatellite markers on the 22 autosomes. Marker allele frequencies were obtained by maximum likelihood estimation using MENDEL [5]. Separate liability classes were assumed for males and females. For two-point linkage analysis, we used MLINK from the LINKAGE package [6]. Here, seven different trait models were taken into account that ranged between multiplicative and additive modes of inheritance. The phenocopy rates in both sexes as well as the disease allele frequency were 0.01 or 0.02 for all models. For multipoint analysis, we used the program GENEHUNTER-MODSCORE [7], which maximizes the LOD score with respect to the penetrances and disease allele frequency. It is a further development of our program GENEHUNTER-IMPRINTING [8] which is based on the original GENEHUNTER version 2.1 [9-11]]. Like GENEHUNTER-IMPRINTING, the program GENEHUNTER-MODSCORE can perform a parametric multipoint linkage analysis under trait models that adequately take imprinting into account. This is done by distinguishing individuals who are heterozygous at the disease locus by the parent who transmitted the disease allele. MOD scores were calculated under four-penetrance imprinting models, as well as under the nonimprinting constraint for which the two heterozygote penetrances are constrained to be equal. This leads to the standard formulation of trait models with three penetrances.

When analyzing the microsatellite data, we selected the 'modcalc single' option under which a separate maximization over trait model parameters is performed for each genetic position of the putative trait locus. The penetrances for both sexes were varied. We used the sex-averaged as well as the sex-specific COGA marker map provided by Stassen [12]. After removing individuals who were untyped or whose trait phenotype was unknown, and one family branch with obvious bilineality, none of the pedigrees had more than 20 effective meioses (2*nonfounders – founders), and so the analysis of the dataset was feasible.

### Affymetrix SNP data

We also used the 11,145 autosomal SNP markers of the Affymetrix GeneChip Human Mapping 10 K Array for a multipoint MOD-score analysis. Starting with the raw (i.e., uncleaned) data, the comprehensive quality control and data conversion was managed by ALOHOMORA [13]. We applied GENEHUNTER-MODSCORE to the resulting dataset in the same way as described above, for the microsatellite data, with imprinting and nonimprinting analyses, and used separate liability classes for males and females. The multipoint analyses were performed in chunks of 100 SNPs (nonimprinting) or 150 SNPs (imprinting). Because the marker density is much higher for the SNPs than for the microsatellites, the number of genetic positions assumed for the disease locus is also much larger for the SNP analysis. In this case, a separate maximization over trait models for each genetic position, as done with the 'modcalc single' option, would have led to excessive computation-time demands, and hence, 'modcalc global' was used for the MOD-score analysis of the SNPs. With this option, the overall maximum over the analyzed genetic region is maximized with respect to the trait-model parameters. Because no sex-specific map was available for the Affymetrix SNPs, we only used the sex-averaged map, according to the annotation file of May 2004. Please note that coordinates from this map are different from the COGA map.

## Results

### Microsatellite markers

Single-marker analysis using LINKAGE yielded suggestive evidence of linkage for two genetic regions. A LOD score of 2.51 was obtained for chromosome 7 at marker D7S1790 (19 cM), and a LOD score of 2.02 for chromosome 10 at marker D10S670 (135 cM). All other two-point LOD scores were below 2 (data not shown).

**Figure 1:**
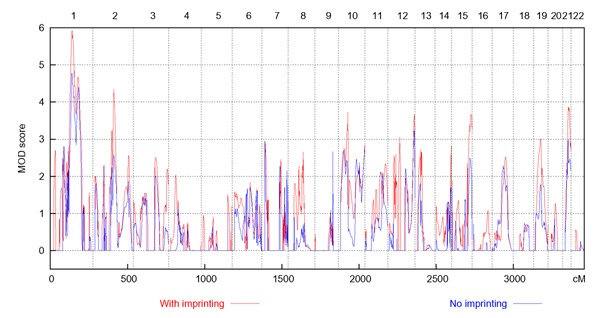
**MOD-score results for microsatellite markers. **Genome-wide plot of the GENEHUNTER-MODSCORE results for the microsatellite markers using the sex-specific map. The red graph shows the MOD scores obtained under four-penetrance trait models that take imprinting into account, and the blue graph shows the MOD scores obtained under the nonimprinting constraint.

A genome-wide plot of the multipoint results obtained with GENEHUNTER-MODSCORE for the microsatellite data is shown in Figure 1, for imprinting trait models with four penetrances (red curve), as well as for standard trait models with three penetrances (blue curve). The plot reflects the analyses performed using the sex-specific map (results for the sex-averaged map not shown). Because a separate maximization over trait models has been performed at every position for the microsatellite data, the MOD score is never below zero. In Table 1, the genetic regions are listed for which the analysis allowing for imprinting yielded a MOD score around or above 3.5 either for the microsatellites (using the sex-specific map) or the Affymetrix SNPs, together with the best-fitting parameters of the trait model. It should be noted that the estimate of the disease allele frequency *p *obtained by a MOD-score analysis has the largest variance of all trait-model parameters. Furthermore, in some cases, the estimated disease allele frequency will be markedly higher than the true value. This is due to the fact that specifying a higher disease allele frequency can compensate for a general model misspecification and hence lead to robustness in a multipoint analysis [14].

With MOD-score analysis under imprinting models, the most prominent linkage peak was obtained for chromosome 1 at 140 cM. When using the sex-averaged map, the MOD score reached 5.29; it further increased to 5.93 with the sex-specific map. The best-fitting penetrances point to a dominant model with nearly complete penetrance in females and a recessive model with strongly reduced penetrance in males. A MOD score of 4.11 (sex-averaged map) and 4.34 (sex-specific map) was obtained for chromosome 2 at 136 cM, with a recessive model in males and a paternal-imprinting model in females. On chromosome 7, at 118 cM, the MOD score was 3.30 for the sex-averaged map; it dropped to 2.46 (at 122 cM) when using the sex-specific map. Two peaks were seen on chromosome 10 with the sex-averaged map, a MOD of 3.27 at 34 cM and a MOD of 3.43 at 61 cM. With the sex-specific map, the first peak drops to 2.75, whereas the second peak increases to 3.73. The best-fitting model at the second peak is additive with complete homozygous-mutant penetrance for males and indicates complete paternal imprinting in females, albeit with a strongly reduced penetrance of 0.16. MOD scores of 3.85 (sex-averaged map) and 3.68 (sex-specific map) were found on chromosome 12 at 172 cM, with the trait model pointing to paternal imprinting. On chromosome 15 at 127 cM, the MOD score reached 3.45 with the sex-averaged map and 3.67 with the sex-specific map; there was evidence for complete paternal imprinting at this locus, with complete penetrance in females but almost no effect in males. Finally, on chromosome 21, a MOD score of 3.76 was obtained for the sex-averaged map at 43 cM and a MOD of 3.86 for the sex-specific map at 38 cM, with the best-fitting model pointing to paternal imprinting.

In order to conclude whether imprinting is present at a certain locus or not, it is possible to look at the difference between the MOD scores obtained under four-penetrance trait models and under standard trait models with three penetrances. This strategy has been proposed in the context of a linkage study of house dust mite allergy [8]. Here, we have observed pronounced differences between imprinting and nonimprinting MOD scores for the loci on chromosomes 2 (4.34 vs. 2.58), 10 (3.73 vs. 2.68), 15 (3.67 vs. 2.49), and 21 (3.86 vs. 2.99), whereas the difference is only moderate at the chromosome 12 locus (3.68 vs. 3.24). A large MOD-score difference was also found for the locus on chromosome 1 (5.93 vs. 4.77); however, the heterozygote penetrances of the best-fitting model differ only slightly for males (Table 1).

### Affymetrix SNP markers

**Figure 2:**
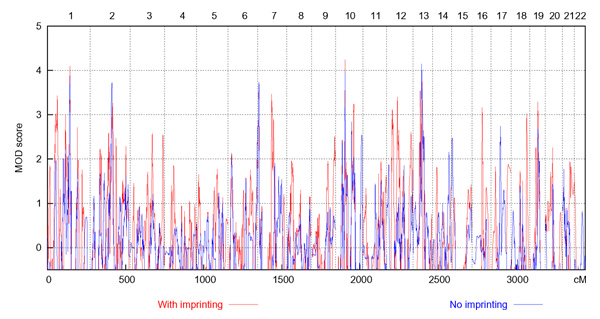
**MOD-score results for Affymetrix SNP markers. **Genome-wide plot of the GENEHUNTER-MODSCORE results for the Affymetrix SNP markers. The red graph shows the MOD scores obtained under four-penetrance trait models which take imprinting into account, and the blue graph shows the MOD scores obtained under the nonimprinting constraint. Here, a sex-averaged map has been used.

Figure 2 shows the genome-wide plot of the multipoint MOD-score results for the Affymetrix SNPs, calculated under imprinting and nonimprinting models. Because the 'modcalc global' option has been used for each chunk of SNPs, the MOD score can fall below zero in this case. Furthermore, at some loci the four-penetrance MOD score stays below the MOD score obtained under the nonimprinting constraint. This artifact occurs when the MOD-score routine falls into a local maximum; the effect is more pronounced for the SNPs ('modcalc global') than for the microsatellites ('modcalc single'). For chromosome 1 at 150 cM, a MOD of 4.10 was found (Table 1); this locus most likely corresponds to the locus showing a peak for the microsatellites at 140 cM. Chromosome 2 yielded a MOD of 3.26 at 147 cM, under a near-recessive model in males and a paternal-imprinting model in females, corresponding to the locus found with microsatellites at 136 cM. On chromosome 7, besides a MOD of 3.46 at 99 cM, which corresponds to the microsatellite result, a second MOD-score peak of 3.66 was obtained at 18 cM, which has correspondence in the microsatellites, too. There was a tendency to maternal imprinting at both chromosome 7 loci. Chromosome 10 showed the highest peak for the SNP markers (MOD = 4.24 at 63 cM), with the best-fitting model corresponding to a near-additive mode of inheritance in males and paternal imprinting in females; this corresponds to the microsatellite result. Chromosome 13 yielded a MOD score of 3.97 at 62 cM, with paternal imprinting in both sexes. A similar result, albeit with a smaller MOD score of 2.73, was also found with the microsatellites (at 37.6 cM).

## Discussion

Alcoholism is most likely governed by a considerable number of genetic factors, and so the contribution of a single gene is small. In addition, it is known that environmental factors play an important role as well. As with almost any complex trait, the mode of inheritance is unknown for alcohol dependence. Therefore, we took the approach of parametric exploratory linkage analysis. In particular, we performed single-marker LOD score analysis using MLINK under seven different trait models, and multipoint MOD-score analysis using GENEHUNTER-MODSCORE. The highest linkage signals were seen on chromosomes 1, 2, 7, 10, 12, 13, 15, and 21. The loci on chromosomes 2, 10, 12, 13, 15, and 21 yielded evidence for paternal imprinting. A tendency to maternal imprinting was observed at two loci on chromosome 7. For the microsatellites, several linkage peaks decreased with the sex-specific map, while others increased; the latter was the case for the loci on chromosomes 10, 15, 21, and most prominently chromosome 1. Daw et al. [15] have shown that using a sex-averaged map instead of the correct sex-specific map can lead to a reduced power to detect linkage and to a strongly increased type I error; therefore, adequate modeling of the recombination between markers is crucial.

Most of the linkage peaks shown in Table 1 were consistently identified with the microsatellite and SNP markers. Remarkably, at most loci the best-fitting trait-model parameters obtained for the two marker sets indicate similar modes of inheritance. Still, overall differences between the microsatellite and SNP results are clearly apparent. These may be due to different marker information content, genotyping errors, or inaccuracies in the genetic maps. Furthermore, it has been shown that falsely assuming linkage equilibrium between closely-spaced markers leads to an increased type I error rate if the markers are in fact in linkage disequilibrium and if there are untyped founders [16]. This problem can arise in the context of SNPs when using multipoint linkage programs such as GENEHUNTER or its derivatives, which assume linkage equilibrium between markers. Still, with the COGA dataset analyzed here, the majority of the founders (62%) had been genotyped for the Affymetrix SNPs. In addition, for many pedigrees, the founders' unknown genotypes can be reconstructed because they have several typed children, which reduces the type I error inflation [16]. Therefore, this effect may be present in our results for the Affymetrix SNPs, but probably not to a large degree.

A MOD-score analysis represents one of the most comprehensive ways to analyze linkage data; we believe this procedure is particularly well suited for the genetic dissection of a complex trait. On the other hand, a MOD-score analysis is clearly exploratory, and so it is difficult to control the type I error. *p*-Values of MOD scores can be obtained by performing simulations for the studied dataset under the null hypothesis of no linkage. However, because *p*-values should be calculated especially for high MOD scores, many replicates need to be analyzed. With the COGA family sample, a substantial amount of computation time was already required for the MOD-score analysis of the original dataset; thus, analyzing many replicates for each of the loci identified here would not be feasible. Instead, we relied on criteria given by Weeks et al. [17] and Hodge et al. [18]. They have found, by performing simulations, that for MOD scores, a critical value of 3, used for LOD scores, should be adjusted by some value in the range of 0.3 to 1.0 to maintain a similar type I error, with the upper boundary being rather conservative. However, these simulations do not account for the additional parameter involved with the imprinting formulation, nor for modeling different penetrances in males and females; therefore, a further adjustment of the critical value is necessary. We put forward that the loci with a MOD score above 3.5 identified in this linkage study of the COGA dataset show at least suggestive evidence for linkage. Furthermore, we conclude that adequately modeling the genotype-phenotype relation is crucial for the genetic mapping of complex traits such as alcohol dependence.

## Abbreviations

COGA: Collaborative Study on the Genetics of Alcoholism

GAW14: Genetic Analysis Workshop 14

SNP: Single-nucleotide polymorphism

## Authors' contributions

KS is the author of the GENEHUNTER-MODSCORE program; he drafted the manuscript and coordinated the realization of the study. RF performed the MOD-score analyses for the microsatellites and curated for the database. FR performed the SNP analyses including quality checks. CW did the analyses with LINKAGE and the selection of the dataset. JD implemented the use of sex-specific maps into GENEHUNTER-MODSCORE. AF contributed allele frequency estimates and selected the genetic maps used. MPB decided on the design of the study and contributed to the discussion of methodology and results. TFW is the group leader and coordinated the collaboration of the study; he decided on the analytical approaches and contributed to the discussion of methodology and results. All authors read and approved the final manuscript.

## Figures and Tables

**Table 1:** MOD scores and estimated trait-model parameters for microsatellites and SNPs^a^

			Penetrances^c^					
				
Chromosome	Position^b^	MOD		*f*_+/+_	*f*_*m*/+_	*f*_+/*m*_	*f*_*m/m*_	*p*
Microsatellites								
1	140.3	5.93	Male	0.0080	0.0100	0.0080	0.1000	0.050
	D1S1631		Female	0.1000	0.9500	0.9500	0.9500	
2	135.9	4.34	Male	0.0450	0.0600	0.0450	0.4900	0.008
	D2S1328		Female	0.0350	0.3900	1.0000	1.0000	
7	20.7	2.94	Male	0.0450	0.1000	0.0450	0.2500	0.010
	D7S1790		Female	0.0500	0.5400	0.0500	1.0000	
7	122.3	2.46	Male	0.0008	0.0020	0.0008	0.0080	0.500
	D7S1799		Female	0.0000	0.0000	0.0000	0.3400	
10	61.3	3.73	Male	0.0000	0.5500	0.5100	1.0000	0.240
	D10S1426		Female	0.0000	0.0000	0.1600	0.1600	
12	171.6	3.68	Male	0.0600	0.1500	0.2100	0.2300	0.020
	D12S1045		Female	0.1300	0.5100	1.0000	1.0000	
13	37.6	2.73	Male	0.0050	0.0050	0.0100	0.0100	4·10^-5^
	D13S325		Female	0.0150	0.1800	0.6600	1.0000	
15	126.6	3.67	Male	0.0080	0.0080	0.0100	0.0100	0.920
	D15S642		Female	0.1100	0.1100	1.0000	1.0000	
21	38.0	3.86	Male	0.0000	0.0500	0.0900	0.0900	0.390
	D21S1440		Female	0.0000	0.0000	0.1000	0.7400	
Affymetrix SNPs								
1	150.0	4.10	Male	0.0500	0.0500	0.0500	0.3600	0.080
	tsc0056040		Female	0.0500	0.5600	0.8300	0.8300	
2	147.2	3.26	Male	0.0450	0.1100	0.0450	0.2700	0.110
	tsc1376918		Female	0.1600	0.4300	1.0000	1.0000	
7	18.3	3.66	Male	0.0250	0.1000	0.0800	0.1300	0.210
	tsc0058614		Female	0.0000	0.0150	0.0150	0.1100	
7	99.2	3.46	Male	0.0000	0.0200	0.0000	0.0500	0.490
	tsc0287517		Female	0.0000	0.7400	0.0000	0.7400	
10	62.9	4.24	Male	0.0000	0.0700	0.0700	0.1100	0.470
	tsc0608195		Female	0.0350	0.0350	0.8700	0.8700	
12	156.1	2.61	Male	0.0450	0.0900	0.0450	0.6000	0.010
	tsc0053128		Female	0.0800	0.0800	0.5800	1.0000	
13	62.0	3.97	Male	0.0500	0.0600	0.1100	0.1300	0.110
	tsc0949707		Female	0.1500	0.1500	1.0000	1.0000	
15		---^d^						
21		---^d^						

^a ^Genetic regions for which the analysis allowing for imprinting yielded a MOD score around or above 3.5 either for the microsatellites (sex-specific map) or the Affymetrix SNPs (sex-averaged map), together with the best-fitting parameters of the trait model.

^b ^The genetic positions of the highest MOD (given in cM, together with the marker closest to the peak) reflect different coordinates for the COGA microsatellite and Affymetrix SNP maps. Therefore, two results given at corresponding positions in the table for microsatellite and SNP markers most likely reflect the same linkage signal, despite possibly different genetic positions for the two marker sets.

^c ^+, the wild-type; *m*, mutant allele, with the paternally inherited allele listed first; *p*, the disease allele frequency. A trait model with *f*_*m*/+ _»*f*_+/m _points to maternal imprinting (i.e., paternal expression), whereas *f*_*m*/+ _«*f*_+/*m *_indicates paternal imprinting (i.e., maternal expression).

^d ^---, In the two genetic regions on chromosomes 15 and 21 mentioned for the microsatellites, MOD scores were below 2 for the Affymetrix SNPs.
